# Predictive models and survival analysis of postoperative mental health disturbances in adult glioma patients

**DOI:** 10.3389/fonc.2023.1153455

**Published:** 2023-04-21

**Authors:** Yi Wang, Jie Zhang, Chen Luo, Ye Yao, Guoyou Qin, Jinsong Wu

**Affiliations:** ^1^Department of Biostatistics, School of Public Health, Fudan University, Shanghai, China; ^2^Department of Neurosurgery, Huashan Hospital, Shanghai Medical College, Fudan University, Shanghai, China; ^3^Neurosurgical Institute, Fudan University, Shanghai, China; ^4^Shanghai Clinical Medical Center of Neurosurgery, Shanghai Municipal Health Commission, Shanghai, China; ^5^Shanghai Key Laboratory of Brain Function and Restoration and Neural Regeneration, Science and Technology Commission of Shanghai Municipality, Shanghai, China; ^6^National Clinical Research Centre for Aging and Medicine, Huashan Hospital, Fudan University, Shanghai, China; ^7^Key Laboratory of Public Health Safety of Ministry of Education, Fudan University, Shanghai, China

**Keywords:** postoperative mental health disturbances, factor analysis, risk factors, glioma, predictive models

## Abstract

**Background and Objectives:**

Patients with primary malignant brain tumors may experience mental health disturbances that can significantly affect their daily life. This study aims to identify risk factors and generate predictive models for postoperative mental health disturbances (PMHDs) in adult glioma patients in accordance with different clinical periods; additionally, survival analyses will be performed.

**Methods:**

This longitudinal cohort study included 2,243 adult patients (age at diagnosis ≥ 18 years) with nonrecurrent glioma who were pathologically diagnosed and had undergone initial surgical resection. Six indicators of distress, sadness, fear, irritability, mood and enjoyment of life, ranging from 0-10, were selected to assess PMHDs in glioma patients in the third month after surgery, mainly referring to the M.D. Anderson Symptom Inventory Brain Tumor Module (MDASI-BT). Factor analysis (FA) was applied on these indicators to divide participants into PMHD and control groups based on composite factor scores. Survival analyses were performed, and separate logistic regression models were formulated for preoperative and postoperative factors predicting PMHDs.

**Results:**

A total of 2,243 adult glioma patients were included in this study. Based on factor analysis results, 300 glioma patients had PMHDs in the third postoperative month, and the remaining 1,943 were controls. Candidate predictors for PMHDs in the preoperative model were associated with age, clinical symptoms (intracranial space-occupying lesion, muscle weakness and memory deterioration), and tumor location (corpus callosum, basal ganglia and brainstem), whereas age, clinical symptoms (nausea and memory deterioration), tumor location (basal ganglia and brainstem), hospitalization days, WHO grade 4, postoperative chemotherapy or radiotherapy and postoperative Karnofsky Performance Scale (KPS) served as important factors in the postoperative model. In addition, the median overall survival (OS) time for glioma patients with PMHDs was 19 months, compared to 13 months for glioblastoma, IDH-wild type (GBM) patients with PMHDs.

**Conclusion:**

The risk factors for PMHDs were identified. These findings may provide new insights into predicting the probability of PMHD occurrence in glioma patients in addition to aiding effective early intervention and improving prognosis based on different clinical stages.

## 1 Introduction

Gliomas are common infiltrative primary brain tumors (1) whose cellular origin remains controversial (2); they can occur in all age groups. The average annual age-adjusted incidence of glioma was 5.95/100,000, with 8.63%, 14.91%, and 76.46% of the population aged 0-14, 15-39, and 40 years or older, respectively (3). Mental disturbances are among the most common causes of death worldwide, accounting for approximately 14.30% of deaths (4), and they are prevalent in patients with glioma (5). Prolonging the survival of patients with gliomas through multiple therapeutic approaches is critical, but the impact of tumor diagnosis and disease progression on patients’ mood alterations and mental health warrants attention (6).

According to previous evidence, the invasive clinical nature of the glioma itself as well as other factors (e.g., treatment regimens) cause direct damage to brain function and may also contribute to neurocognitive changes (7–9) and psychological disorders (10). Approximately 19-80% of glioma patients undergoing oncologic surgery experience perioperative and peritumoral infarctions, which are correlated with postoperative neurologic dysfunction and impaired function (11). Mental health disturbances have a dramatic influence on life after cancer diagnosis, including cancer treatment and ongoing care, which poses substantial challenges and burdens for both patients and their family members who may supply them with supportive care services (12). Patients with glioma are more susceptible to moderate-to-severe depressive symptoms than cancer-free patients (13). The 6-month prevalence of clinical depression in brain tumor patients is approximately 20%, while up to 60% of patients may be affected by personality changes (14). The suicide incidence among cancer patients is 20% higher than that among the general population; therefore, the early period after disease diagnosis is a critical intervention phase for mental health issues (15).

Fewer papers than expected are currently available on mental health in glioma patients, and most available reports are limited by smaller sample sizes (16) in addition to focusing on single disorders such as depression (17). Explicit identification of potential risks for multiple indicators of mental health disturbances is urgently needed to further advance disease management and prevention. A study with a larger and more balanced population based on all pathological grades is encouraged. Although the etiological basis of glioma and the mechanisms underlying mental health disturbances remain obscure (18), the strategy of identifying clinical risk factors associated with their development to predict patient outcomes is feasible (19) and can be implemented to assist clinicians with earlier intervention, improve clinical diagnosis, and decrease the incidence of postoperative mental health disturbances (PMHDs).

Herein, we investigated the risk factors for PMHDs in glioma patients who had undergone tumor resection by collecting detailed demographic data and clinical information from the preoperative and postoperative stages to establish accurate prediction models for PMHDs and to compare the discrimination of survival between participants in the PMHD and control groups. The intent of predictive models is to identify target populations requiring priority attention, provide individualized early intervention and treatment strategies and support optimal evidence-based disease management, as well as better assess prognosis and improve the quality of patient survival.

## 2 Materials and methods

### 2.1 Patients

**Figure 1:**
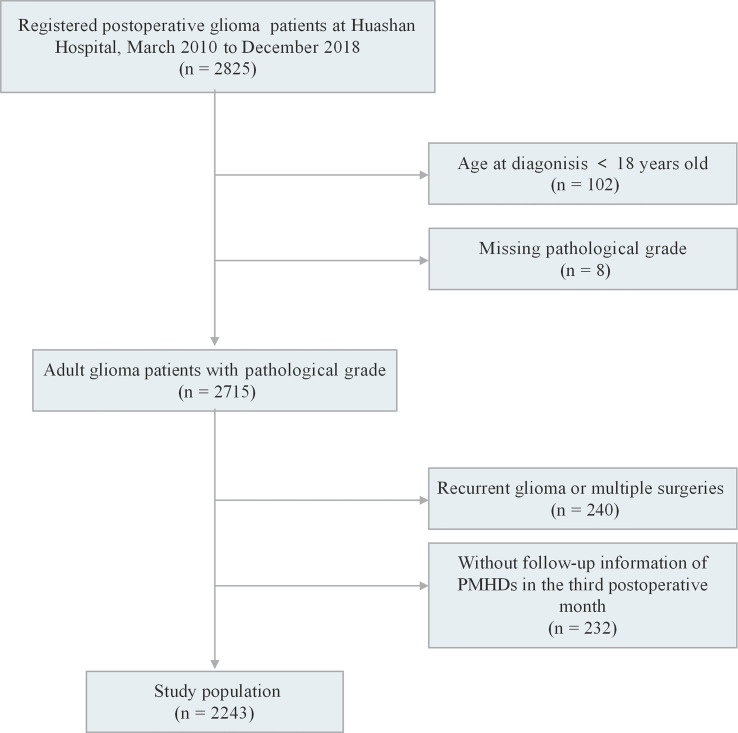
Details of study recruitment. PMHDs, postoperative mental health disturbances.

We recruited 2,825 participants who were pathologically diagnosed with glioma and had undergone maximal safe resection at Huashan Hospital from March 2010 to December 2018. The exclusion criteria included the following (1): aged <18 years old at diagnosis; (2) missing WHO grade; (3) recurrent glioma or multiple surgeries; and (4) no follow-up information of PMHDs in the third postoperative month. A total of 2,243 patients met all criteria for inclusion in the study (**Figure 1**). The study protocol was approved by the ethics review committee of the Huashan Hospital Affiliated to Fudan University (receiving ethics committee number KY2015-256). Written informed consent was signed by all participants of the Central Nervous System Disease Tissue Bank at Huashan Hospital.

### 2.2 Clinical data

Meatal health disturbance in the third postoperative month consisted of six indicators: distress, sadness, fear, irritability, mood, and enjoyment of life. Data for all indicators were collected at the third month postoperative follow-up. The selection of indicators referred to the M.D. Anderson Symptom Inventory Brain Tumor Module (MDASI-BT) (20) and the final list of symptoms based on content validity index (CVI) (21). The MDASI-BT is a patient-reported site-specific brain tumor module that evaluates the severity of symptoms experienced by cancer patients and the potential impact of those symptoms on daily life. The final list of symptoms was identified by the team that developed the MDASI-BT after conducting a CVI analysis of multiple symptoms prior to developing the scale. Each indicator refers to an eleven-point scale ranging from the lowest degree (0) to the highest degree (10) in one-point increments, with 0 being “not present” and 10 being “as bad as you can imagine” in the last 24 hours. Basic demographic information, clinical data and regular follow-up information of the participants were collected from the hospital’s electronic medical record system. The preoperative phase consisted of 40 variables related to demographic information, clinical symptoms, disease history, and tumor location. It should be mentioned that the tumor location variables are not independent of each other; for example, multifocal gliomas could involve multiple tumor locations simultaneously, whereas a single tumor may also involve different brain lobes. A series of 14 variables were incorporated in the postoperative phase, including WHO grade, molecular indicators and postoperative scale scores (**Table 1**).

**Table 1:** Demographic and clinical data of patients with postoperative mental health disturbances and controls.

Demographic and Clinical Data	PMHD† (n = 300)	Controls (n = 1943)	t/Z/χ^2^	*p* value	Adjusted *p* value
Preoperative Period					
**Age at Diagnosis (Years)**	51.96 ± 13.65, 18.00-79.00	45.31 ± 12.78, 18.00-82.00	t = 7.92	2.57×10^-14**^	1.39×10^-12**^
**Sex male/female**	182/118	1126/817	χ^2 ^= 0.79	0.37	1.00
**Illness Duration (Months)**	5.20 ± 13.45, 0.10-120.00	5.53 ± 14.96, 0.05-240.00	t = -0.34	0.73	1.00
**Headache yes/no**	104/196	738/1205	χ^2 ^= 1.22	0.27	1.00
**Dizziness yes/no**	38/262	281/1662	χ^2 ^= 0.69	0.41	1.00
**Nausea yes/no**	4/296	74/1869	χ^2 ^= 4.74	0.03	1.00
**Vomiting yes/no**	16/284	123/1820	χ^2 ^= 0.44	0.51	1.00
**Epilepsy yes/no**	12/288	154/1789	χ^2 ^= 5.84	0.02	0.84
**Incidental Space Occupying Lesion or Incidental Tumor† yes/no**	7/293	125/1818	χ^2 ^= 7.89	4.98×10^-3*^	0.27
**Impaired Consciousness yes/no**	43/257	383/1560	χ^2 ^= 4.89	0.03	1.00
**Muscle Twitching yes/no**	44/256	401/1542	χ^2 ^= 5.83	0.02	0.85
**Muscle Weakness yes/no**	74/226	226/1717	χ^2 ^= 38.11	6.68×10^-10**^	3.61×10^-8**^
**Limb Numbness yes/no**	31/269	123/1820	χ^2 ^= 6.51	0.01	0.58
**Speech Disorder yes/no**	37/263	153/1790	χ^2 ^= 6.66	0.01^*^	0.53
**Memory Deterioration yes/no**	28/272	73/1870	χ^2 ^= 18.79	1.46×10^-5*^	8.10×10^-4*^
**Slow Reaction yes/no**	15/285	37/1906	χ^2 ^= 11.00	9.12×10^-4*^	0.05
**Visual Impairment yes/no**	16/284	77/1866	χ^2 ^= 1.23	0.27	1.00
**Lethargy yes/no**	4/296	9/1934	Z = 1.73	0.08	1.00
**Family History of Glioma yes/no**	0/294	1/1911	Z = 0.00	1.00	1.00
**Respiratory Diseases yes/no**	0/200	0/1491	–	–	–
**Digestive Diseases yes/no**	1/200	1/1490	Z = 1.22	0.22	1.00
**Urinary System Diseases yes/no**	0/200	2/1473	Z = 0.00	1.00	1.00
**Hematological Disorders yes/no**	0/199	0/1475	–	–	–
**Endocrine Diseases yes/no**	0/182	1/1426	Z = 0.00	1.00	1.00
**Cardiovascular Diseases yes/no**	0/198	3/1462	Z = 0.00	1.00	1.00
**History of Surgery Unrelated to Glioma yes/no**	9/285	24/1890	Z = 2.13	0.03	1.00
**History of Head Trauma yes/no**	1/292	4/1906	Z = 0.66	0.51	1.00
**Chemicals or Special Drug Exposure yes/no**	0/287	2/1880	Z = 0.00	1.00	1.00
**Tumor Location (Frontal) yes/no**	154/146	1055/888	χ^2 ^= 0.92	0.34	1.00
**Tumor Location (Parietal) yes/no**	51/249	258/1685	χ^2 ^= 3.03	0.08	1.00
**Tumor Location (Occipital) yes/no**	26/274	114/1829	χ^2 ^= 3.48	0.06	1.00
**Tumor Location (Temporal) yes/no**	93/207	601/1342	χ^2 ^= 5.70×10^-4^	0.98	1.00
**Tumor Location (Insular) yes/no**	23/277	150/1793	χ^2 ^= 1.04×10^-3^	0.97	1.00
**Tumor Location (Corpus Callosum) yes/no**	18/282	60/1883	χ^2 ^= 6.57	0.01	0.56
**Tumor Location (Thalamus) yes/no**	8/292	29/1914	Z = 1.46	0.14	1.00
**Tumor Location (Basal Ganglia) yes/no**	12/288	25/1918	χ^2 ^= 11.79	5.95×10^-4*^	0.03
**Tumor Location (Brainstem) yes/no**	4/296	3/1940	Z = 2.66	0.01^*^	0.43
**Tumor Location (Cerebellum) yes/no**	5/295	31/1912	Z = 0.24	0.81	1.00
**Tumor Location (Ventricle) yes/no**	8/292	49/1894	χ^2 ^= 0.02	0.88	1.00
**Karnofsky Score †‡ (%)**	82.94 ± 13.16, 30.00-100.00	84.76 ± 12.40, 40.00-100.00	t = -2.24	0.03	1.00
Postoperative Period					
**WHO Grade 1/2/3/4**	3/66/32/199	36/699/247/961	χ^2 ^= 31.15	7.90×10^-7**^	4.27×10^-5**^
**WHO Grade 1 yes/no**	3/297	36/1907	χ^2 ^= 1.11	0.29	1.00
**WHO Grade 2 yes/no**	66/234	699/1244	χ^2 ^= 22.58	2.01×10^-6**^	1.08×10^-4*^
**WHO Grade 3 yes/no**	32/268	247/1696	χ^2 ^= 1.00	0.32	1.00
**WHO Grade 4 yes/no**	199/101	961/982	χ^2 ^= 29.63	5.22×10^-8**^	2.82×10^-6**^
**IDH-1/IDH-2+† yes/no**	35/105	731/892	χ^2 ^= 21.07	4.44×10^-6**^	2.16×10^-4*^
**1p/19q Codeletion† yes/no**	63/46	473/395	χ^2 ^= 0.43	0.51	1.00
**MGMT+† yes/no**	89/166	737/630	χ^2 ^= 31.08	2.47×10^-8**^	1.34×10^-6**^
**TERT+† yes/no**	63/46	473/395	χ^2 ^= 0.43	0.51	1.00
**EGFR+† yes/no**	5/1	97/28	χ^2 ^= 0.11	0.74	1.00
**GFAP+† yes/no**	277/20	1875/50	χ^2 ^= 14.43	1.45×10^-4*^	0.01^*^
**OLIG2+† yes/no**	194/97	1564/341	χ^2 ^= 37.66	8.44×10^-10**^	4.56×10^-8**^
**P53+† yes/no**	106/187	931/968	χ^2 ^= 16.81	4.13×10^-5*^	2.21×10^-3*^
**Ki-67 ≥ 20% yes/no**	64/231	310/1570	χ^2 ^= 4.85	0.03	1.00
**Chemotherapy/Radiotherapy yes/no**	226/74	1619/324	χ^2 ^= 11.37	7.46×10^-4*^	1.22×10^-6**^
**Hospitalization Days**	21.01 ± 9.75, 3.00-67.00	17.59 ± 7.86, 2.00-131.00	t = 5.72	2.27×10^-8**^	0.04
**Karnofsky Score †‡ (%)**	75.33 ± 18.09, 20.00-100.00	92.37 ± 11.33, 40.00-100.00	t = -15.49	7.69×10^-41**^	4.15×10^-39**^
**ECOG Score†**	1.54 ± 0.77, 0.00-4.00	0.68 ± 0.65, 0.00-3.00	t = 18.00	8.81×10^-52**^	4.76×10^-50**^

* Continuous data are shown as the mean ± SD, minimum and maximum values in patients with postoperative mental health disturbances (PMHDs) and controls with statistical significance based on a two-sample t test. Categorical data differences in patients with PMHDs and controls are represented with statistical significance based on the chi-squared test (χ^2^ & *p*) and Fisher’s exact test (Z & *p*). *: *p* < 0.01, **: *p* < 1×10^-4^.

† The incidental space occupying lesion or incidental tumor were discovered incidentally through seeking medical attention for an accidental injury or physical examination. PMHD, postoperative mental health disturbances; IDH-1/IDH-2**+**, isocitrate dehydrogenase 1 or isocitrate dehydrogenase 2 mutation; 1p/19q codeletion, complete deletion of both the short arm of chromosome 1 (1p) and the long arm of chromosome 19 (19q); MGMT+, O-6-methylguanine-DNA methyltransferase promoter methylation; TERT+, telomerase reverse transcriptase promoter mutation; EGFR+, epidermal growth factor receptor mutation; GFAP**+**, glial fibrillary acidic protein positive; OLIG2**+**, oligodendrocyte transcription factor 2 overexpression; p53**+**, tumor suppressor gene p53 mutation; Ki-67, cell proliferation antigen Ki-67. The Karnofsky score refers to an eleven-point rating scale that ranges from normal functioning (100%) to death (0%) in 10% increments. The ECOG score is based on a scoring system that measures the extent to which cancer affects a patient’s daily living abilities (performance status) on a scale ranging from 0 (fully active) to 5 (dead).

‡ 299 patients with PMHDs and 1,932 controls attended preoperative KPS score tests, while 287 patients with PMHDs and 1845 controls participated in the postoperative KPS score tests.

### 2.3 Statistical analysis

The Kaiser−Meyer−Olkin (KMO) test and Bartlett’s test of sphericity were conducted to investigate the sampling adequacy for utilizing factor analysis and the homogeneity of the data, respectively. A sample size with a KMO value greater than 0.5 is generally considered to be sufficient to perform factor analysis, and a *p* value of less than 0.05 in Bartlett’s test demonstrates data homogeneity. Principal component analysis and eigenvalues greater than 1.00 were used to extract the factors, and the maximum variance method was used as the rotation method. The least absolute shrinkage and selection operator (LASSO) was applied to sequence variables in different clinical periods and filter out the top 15 variables. The LASSO method, which was proposed by statistics professor Robert Tibshirani in 1996, is a high-dimensional data analysis method for performing regularization on data models and ranking the importance of variables (22). Continuous data are shown as the mean ± standard deviation and minimum - maximum values, and Student’s t test was used for statistical analysis to compare the two groups. The statistical significance of the differences in categorical data between PMHD patients and control subjects was determined by the chi-squared test and Fisher’s exact test. Prediction models were tested for multicollinearity using the variance inflation factor (VIF). Pearson’s correlation coefficient was used to measure linear correlation between continuous variables, while Cramer’s v was applied to measure the strength of association between categorical variables (23). Kaplan−Meier survival curves and log-rank tests were applied to compare the survival distributions between the two groups. Overall survival (OS) time was defined as the period elapsing between the date of the first glioma surgery and date of death. The sensitivity and specificity of the PMHD prediction models developed by implementing the logistic regression method were evaluated with receiver operating characteristic (ROC) curves. In addition, DeLong’s test was conducted to show whether the area under the ROC curves (AUCs) of the two models were significantly different. Statistical significance was denoted by *p* ≤ 0.05. SAS 9.4 and R version 4.2.1 were used for statistical analysis.

## 3 Results

### 3.1 Factor analysis

**Figure 2:**
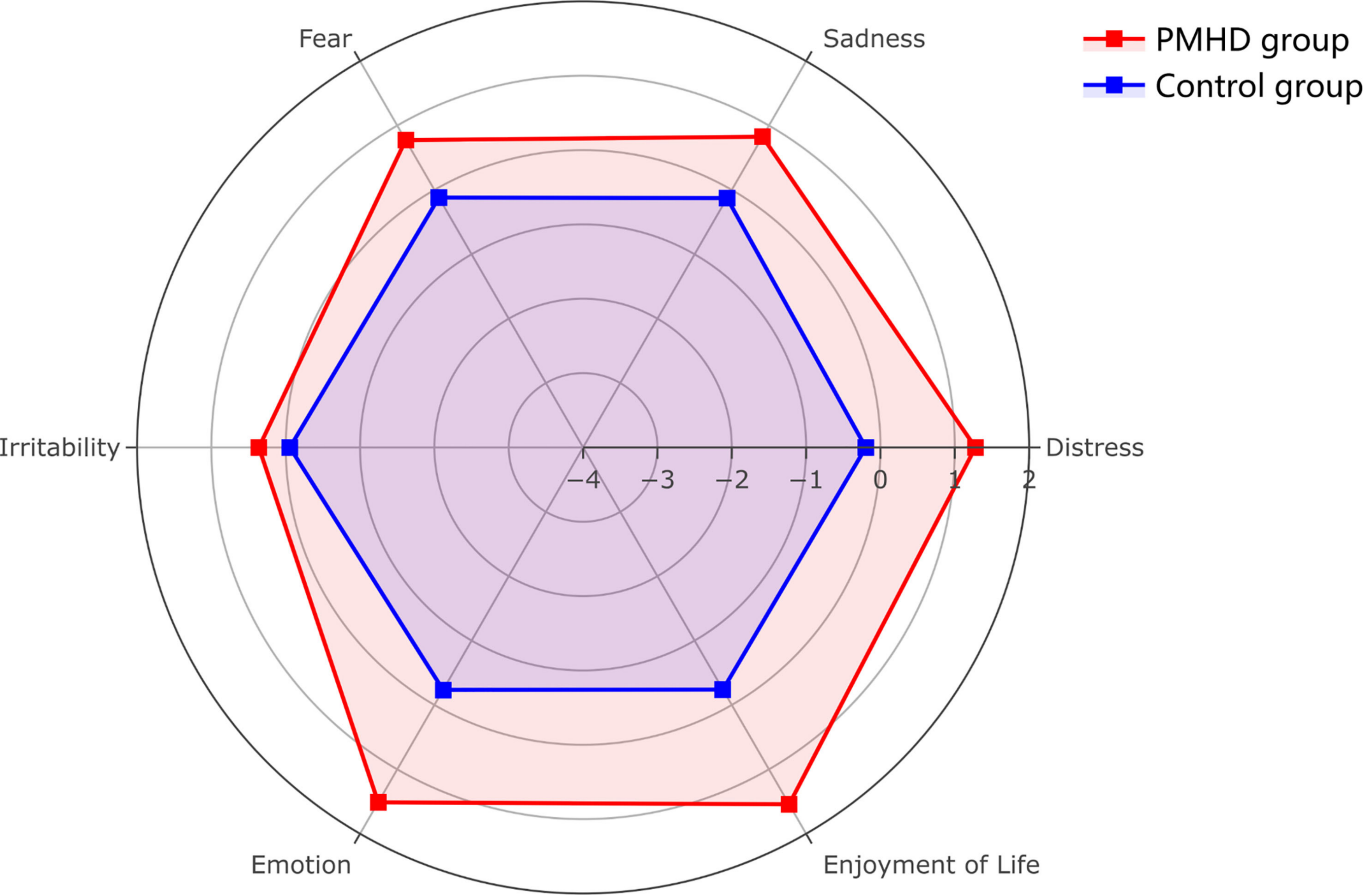
Radar plot of six indicators of postoperative mental health disturbances in the PMHD and control groups. The figure shows the mean values of the six indicators after standardization (patients with PMHDs in red, controls in blue).

A total of 2,243 patients were enrolled in this study (**Figure 1**). The factor analysis of the six indicators that were assessed in glioma patients at the third postoperative month showed a KMO value of 0.69 and a Bartlett test *p* value of less than 0.05, indicating the feasibility of factor analysis. The two components with eigenvalues greater than 1.00 were finally extracted through factor analysis, which could explain 37.08% and 33.90% of the variance, respectively. The composite scores of patients’ psychiatric conditions were calculated based on these two components, and all patients were divided into two groups: 300 (13.37%) patients were assigned to the PMHD group, and the remaining 1,943 (86.63%) patients were assigned to the control group. The mean of standardized values of the six indexes between the two groups with significant differences were displayed in **Figure 2**, suggesting that patients in the control group performed better than those in the PMHD group in terms of postoperative mental health disturbances.

### 3.2 Patient characteristics

A total of 2,243 adult glioma patients were predominantly male (1,308/2,243; 58.31%), with a mean age at diagnosis of 46.20 years (standard deviation = 13.09). In accordance with the composite score of the factor analysis, 300 (13.37%) of these patients suffered from mental health disturbances at the third month postoperative follow-up, while the remaining 1943 (86.63%) cases served as a control group with relatively favorable mental status. Patients in the PMHD group were significantly older than those in the control group (mean [SD] age, 51.96 [13.65] vs. 45.31 [12.78] years, t = 7.92, *p* < 0.001). Patients with gliomas located in specific positions, such as the corpus callosum (18/300 [6.00%] vs. 60/1943 [3.09%], χ^2 = ^6.57, *p* = 0.01), basal ganglia (12/300 [4.00%] vs. 25/1943 [1.29%], χ^2 = ^11.79, *p* < 0.001), and brainstem (4/300 [1.33%] vs. 3/1943 [0.15%], Z = 2.66, *p* = 0.01), were more prone to PMHDs. In addition, 66.33% (199/300) and 49.46% (961/1943) patients had WHO grade 4 gliomas in the PMHD and control groups, respectively. The detailed clinical variables and the respective statistical results of the univariate analysis for the preoperative and postoperative phases are tabulated in **Table 1**.

### 3.3 Prediction models and survival analysis

Multiple variables were found to be significantly different between the PMHD and control groups by a univariate analysis which included all variables. Therefore, the importance of the variables was ranked separately based on different study populations using the LASSO method, and the top 15 variables were selected to construct different models for predicting PMHDs. Both models in **Table 2** are for all glioma patients, whereas the two postoperative models in **Table 3** are for glioblastoma, IDH-wildtype (GBM) patients and diffuse astrocytoma, IDH-mutant (DA) patients, respectively. To improve the generalizability of the models, molecular indicators were not included in the postoperative model as well as in the predictive model for DA. In addition, there was a strong correlation between Karnofsky Performance Scale (KPS) score and Eastern Cooperative Oncology Group (ECOG) score, with a Pearson’s correlation coefficient of -0.81, and the variable ECOG was excluded from the screening of variables.

**Table 2:** Multivariable logistic regression model for predicting postoperative mental health disorders in glioma patients.

Variables	Preoperative Model Odds Ratio (95% CI)	*p* value	Postoperative Model Odds Ratio (95% CI)	*p* value
Preoperative Period				
Age (Years at Diagnosis)	1.03 (1.02, 1.04)	1.23×10^-9**^	1.02 (1.01, 1.03)	6.60×10^-4*^
Headache yes/no	0.90 (0.69, 1.18)	0.44		
Nausea yes/no	0.35 (0.12, 1.01)	0.05	0.27 (0.09, 0.81)	0.02^*^
Incidental Space Occupying Lesion yes/no	0.44 (0.20, 0.96)	0.04^*^	0.44 (0.19, 1.01)	0.05
Muscle Weakness yes/no	1.73 (1.26, 2.38)	7.68×10^-4*^	1.20 (0.83, 1.73)	0.34
Limb Numbness yes/no	1.55 (0.99, 2.40)	0.05	1.23 (0.73, 2.09)	0.44
Speech Disorder yes/no	1.40 (0.93, 2.10)	0.11	1.27 (0.80, 2.01)	0.32
Memory Deterioration yes/no	1.82 (1.09, 3.03)	0.02^*^	1.79 (1.02, 3.16)	0.04^*^
Slow Reaction yes/no	1.36 (0.68, 2.69)	0.38		
Lethargy yes/no	2.84 (0.83, 9.72)	0.10	0.61 (0.13, 2.82)	0.52
Tumor Location (Temporal Lobe) yes/no	0.91 (0.69, 1.21)	0.53		
Tumor Location (Corpus Callosum) yes/no	1.81 (1.01, 3.21)	0.04^*^	1.39 (0.69, 2.79)	0.36
Tumor Location (Thalamus) yes/no	2.07 (0.89, 4.85)	0.09		
Tumor Location (Basal Ganglia) yes/no	3.30 (1.56, 6.99)	1.85×10^-3*^	4.11 (1.82, 9.28)	6.87×10^-4*^
Tumor Location (Brainstem) yes/no	5.92 (1.26, 27.87)	0.02^*^	10.19 (1.87, 55.53)	0.01^*^
Postoperative Period				
WHO Grade			——	0.12
WHO Grade 2 (Reference)			——	——
WHO Grade 1			0.90 (0.22, 3.70)	0.89
WHO Grade 3			1.53 (0.91, 2.58)	0.11
WHO Grade 4			1.59 (1.08, 2.35)	0.02^*^
Hospitalization Day			1.02 (1.00, 1.04)	0.01^*^
Chemotherapy/ Radiotherapy yes/no			0.67 (0.45, 0.99)	0.04^*^
Karnofsky Score (%)			0.94 (0.93, 0.95)	2.72×10^-42**^

*: *p* < 0.05, **: *p* < 1.00×10^-4^.

**Table 3:** Multivariable logistic regression model for predicting postoperative mental health disorders in different subgroups of glioma patients.

Variables	Glioblastoma, IDH-wildtype Odds Ratio (95% CI)	*p* value	Diffuse Astrocytoma, IDH-mutant Odds Ratio (95% CI)	*p* value
Preoperative Period				
**Age (Years at Diagnosis)**	1.02 (0.99, 1.05)	0.21	1.06 (1.01, 1.11)	0.02^*^
**Illness Duration (Months)**			0.91 (0.78, 1.06)	0.24
**Headache yes/no**	1.05 (0.47, 2.35)	0.91	1.35 (0.35, 5.11)	0.66
**Dizziness yes/no**			1.38 (0.30, 6.36)	0.68
**Epilepsy yes/no**			2.70 (0.39, 18.75)	0.31
**Impaired Consciousness yes/no**			0.90 (0.20, 4.00)	0.89
**Muscle Twitching yes/no**	1.28 (0.36, 4.53)	0.70		
**Muscle Weakness yes/no**	1.51 (0.65, 3.51)	0.34	2.79 (0.42, 18.66)	0.29
**Limb Numbness yes/no**	2.31 (0.61, 8.71)	0.21		
**Speech Disorder yes/no**	1.49 (0.54, 4.11)	0.45		
**Memory Deterioration yes/no**	4.28 (1.34, 13.65)	0.01^*^	6.36 (0.59, 68.81)	0.13
**Visual Impairment yes/no**	0.74 (0.15, 3.76)	0.72	6.27 (1.47, 83.27)	0.02^*^
**Parietal lobe Glioma yes/no**			0.39 (0.04, 3.69)	0.41
**Occipital Lobe Glioma yes/no**	3.30 (1.18, 9.21)	0.02^*^		
**Temporal Lobe Glioma yes/no**	1.69 (0.79, 3.62)	0.18	0.42 (0.10, 1.77)	0.38
**Insular Glioma yes/no**			0.98 (0.15, 6.50)	0.98
**Thalamic Glioma yes/no**	2.62 (0.43, 15.88)	0.29		
Postoperative Period				
**WHO Grade**			——	0.40
WHO Grade 2 (Reference)			——	——
WHO Grade 3			1.46 (0.35, 6.15)	0.60
WHO Grade 4			2.77 (0.64, 12.04)	0.18
**MGMT+ yes/no**	1.17 (0.55, 2.51)	1.17		
**TERT+ yes/no**	0.75 (0.36, 1.52)	0.42		
**Hospitalization day**	1.02 (0.98, 1.05)	0.34	1.10 (1.03, 1.18)	4.80×10^-3*^
**Karnofsky Score (%)**	0.93 (0.91, 0.95)	2.21×10^-11**^	0.93 (0.90, 0.96)	1.33×10^-5*^

*: *p* < 0.05, **: *p* < 1.00×10^-4^.

Further logistic regression analysis revealed that the age at diagnosis (preoperative model: odds ratio [OR], 1.03; 95% confidence interval [CI], 1.02-1.04; *p* < 0.001; postoperative model: OR, 1.02; 95% CI, 1.00-1.03; *p* < 0.001) was significantly different in the two models (**Table 2**). Additionally, clinical symptoms of incidental findings of intracranial space occupying lesion (OR, 0.44; 95% CI, 0.20-0.96; *p* = 0.04), muscle weakness (OR, 1.73; 95% CI, 1.26-2.38; *p* < 0.001) and memory deterioration (OR, 1.82; 95% CI, 1.09-3.03; *p* = 0.02) were critical influencing factors in the preoperative model. Tumors growing in specific locations, such as the corpus callosum (OR, 1.81; 95% CI, 1.01-3.21; *p* = 0.04), basal ganglia (OR, 3.30; 95% CI, 1.56-6.99; *p* = 1.85×10^-3^), and brainstem (OR, 5.92; 95% CI, 1.26-27.87; *p* = 0.02), were also significantly associated with PMHDs. In the multivariate postoperative model, nausea (OR, 0.27; 95%CI, 0.09-0.81; *p* = 0.02), memory deterioration (OR, 1.79; 95% CI, 1.02-3.16; *p* = 0.04), basal ganglia glioma (OR, 4.11; 95% CI, 1.82-9.28; *p* < 0.001), brainstem glioma (OR, 10.19; 95% CI, 1.87-55.53; *p* = 0.01), WHO grade 4 (OR, 1.59; 95% CI, 1.08-2.35; *p* = 0.02), hospitalization day (OR, 1.02; 95% CI, 1.00-1.04; *p* = 0.01), and postoperative Karnofsky Performance Scale (KPS) score (OR, 0.94; 95% CI, 0.93-0.95; *p* < 0.001) were regarded as pivotal predictors of PMHDs.

**Figure 3:**
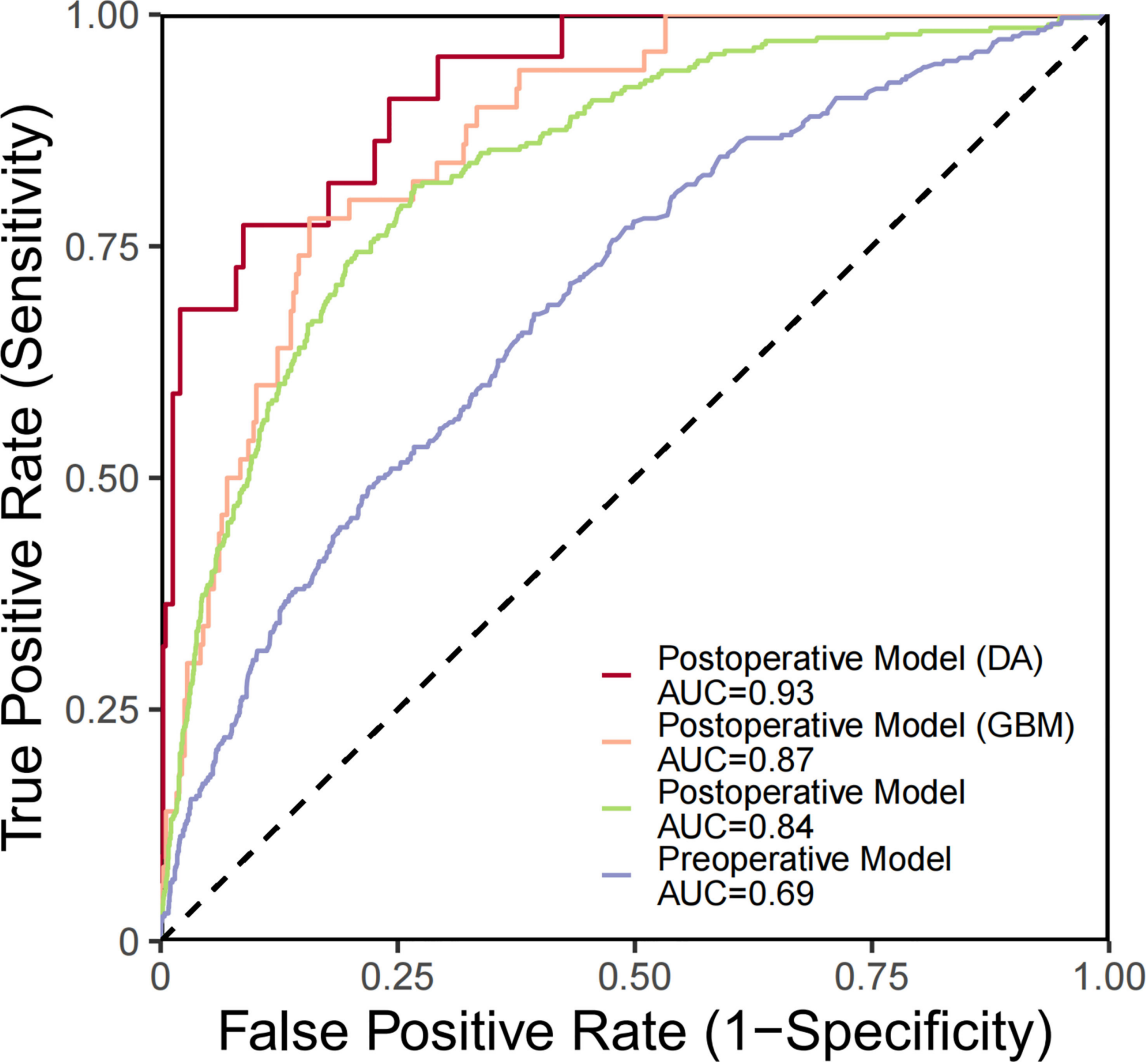
ROC curves generated for PMHD prediction with different clinical periods and subgroups. DA, diffuse astrocytoma, IDH-mutant; GBM, glioblastoma, IDH-wildtype.

Predictive models for GBM and DA were specifically investigated (**Table 3**). ROC curve analysis was utilized to validate the performance of the PMHD prediction models which were stratified by different clinical periods or subgroups (**Figure 3**). The AUCs of the multivariate prediction models varied from 0.69 to 0.93, with the highest AUC being for DA. Nevertheless, the postoperative model outperformed the model constructed by preoperative variables only, with a *p* value of less than 0.05 for the two curves by DeLong’s test indicating a significant difference. VIF was used to detect the severity of multicollinearity in the multivariate logistic prediction models, and the VIFs of the variables were all less than 1.80 (**eTable 1**). A simplified version of the prediction model with an AUC of 0.82 was also constructed for the purpose of model generalization (**eTable 2**).

**Figure 4:**
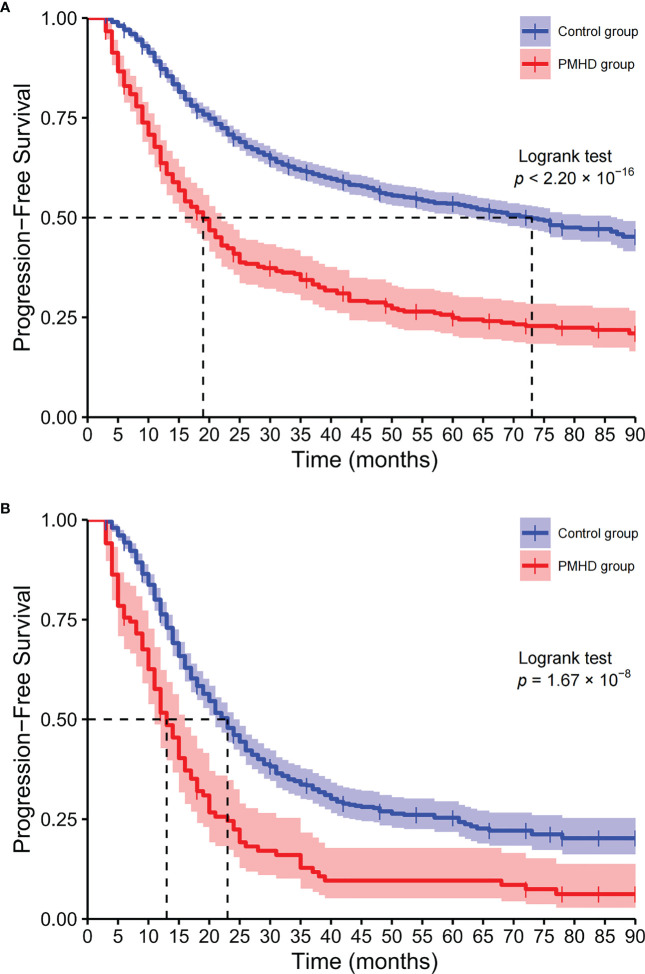
Kaplan−Meier curves of overall survival. Kaplan-Meier plots of overall survival for all glioma patients **(A)** and glioblastoma, IDH-wildtype **(B)** patients (PMHD group in red, control group in blue).

Among all glioma patients, the median OS was 19 and 73 months in the PMHD and control groups, with the 5-year survival rates of 24.88% and 53.51%, respectively (**Figure 4**). While in GBM patients, the median OS was 13 and 23 months and the 5-year survival rates were 9.61% and 25.36% in the PMHD and control groups, respectively. Additionally, in IDH-mutated WHO grade 4 diffuse astrogliomas, the median OS was significantly shorter in patients with PMHDs than in controls (14 vs. 46 months, P < 0.001). In low-grade gliomas (LGG) containing WHO grades 1 and 2, the 5-year survival rate for patients with PMHDs was found to be 73.96%, lower than the 86.33% for controls.

## 4 Discussion

This longitudinal cohort study enrolled 2,243 glioma patients at the Huashan Hospital over an 8-year period, providing demographic information and clinical data on a sizeable sample involving all WHO grades. In our study, separate preoperative and postoperative predictive models were constructed to pinpoint the potential clinical risk factors for mental health disturbances in glioma patients after surgical resection. Postoperative predictive models specifically targeting the GBM and DA subgroups were also performed. Survival analysis of multiple datasets revealed that patients with mental health disturbances had a worse prognosis and shorter survival times than did controls; Glioma patients with PMHDs had a median survival time of 19 months, whereas the median survival time was only 13 months for GBM patients with PMHDs. The current study aimed to accurately predict the probability of mental health disturbances in the short term (within three months) postoperatively from different clinical stages, to clearly identify special populations that need focused attention and to deliver promising clinical reference values for evidence-based early intervention and cancer risk management.

Mental health disturbances were common neuropsychiatric complications and comorbidities in patients with brain tumors (24, 25). The incidence of mental health disturbances in this study was relatively underestimated at only 13.37%; this was probably attributable to our appraisement of glioma patients’ mental status in the third postoperative month. Patients who failed to complete the MDASI-BT in the third postoperative month due to missed visits or death were excluded from the study. Moreover, some patients experienced partial progress in physical function and relative improvement in mental status after enduring the risks inherent in brain tumor surgery and prolonged postoperative rehabilitation. The stigma associated with mental health disorders, influenced by cultural and contextual value systems (26), was also a potential reason for the relatively low reporting rate. Additionally, it should be noted that mental health disturbances are not equivalent to psychiatric disorders. Therefore, the incidence rate of PHMDs in this study should not be directly compared to other statistics in the literature. Glioma patients generally suffer from the mental stress of a poor prognosis, considerable financial pressure, the symptom burden of the disease and decreased workforce participation (27) throughout the disease trajectory. The tumor and its treatment options may affect the brain with far-reaching mental health consequences. As the mental symptoms progress, the need for care support among glioma patients gradually increases, imposing a heavy burden on caregivers, families, the healthcare system and society (28). Therefore, identifying risk factors that contribute to mental health disturbances and providing early and effective interventions are critical for improving the quality of life of glioma patients (29, 30).

The incidence of glioma varies with age (31), and advanced age is broadly recognized as a predictor of poor prognosis in glioma patients (32, 33); this was consistent with the relevant findings in our study. Several studies (34, 35) have reported that glioma patients with global cerebral manifestations, focal neurologic deficits and neurocognitive dysfunction will endure unfavorable life effects; these results support our research findings that glioma patients with clinical symptoms of nausea, muscle weakness, memory deterioration and visual impairment are more prone to experiencing mental health disturbances. Visual impairment can be caused by lesions in brain regions involved in the visual pathway, such as the occipital, parietal and temporal lobes, as well as factors such as high intra-cranial pressure. In addition, it was noteworthy that the vast majority of glioma patients experienced partial relief of their neuropsychiatric symptoms after maximal safe tumor resection (36, 37). Thus, preoperative neurological deficits may be potentially valuable for early detection of disease, but they were not equivalent to the patient’s postoperative mental status and could not directly influence PMHDs. Intracranial space occupying lesion (ICSOL) or intracranial tumor, discovered incidentally through accidental injury seeking medical attention or routine physical examination, was a potential protective factor for the occurrence of PMHD in glioma patients. Incidental findings of brain abnormalities were not relevant to the purpose of this examination (38). The symptoms associated with glioma were less evident in these patients at the time of brain imaging, demonstrating the value of early detection of latent disease and facilitating early intervention.

The anatomic location of gliomas considerably influences prognosis and therapeutic options. Gliomas growing in specific regions undoubtedly impose immense challenges for surgical resection and carry potential risks and complications that may induce neurological impairments. Lesions of the corpus callosum, the largest bundle of white matter fibers that extensively connect the left and right hemispheres of the brain, may affect the exchange of information between the bilateral hemispheres and lead to mental health disturbances and cognitive deficits (39). The basal ganglia, located beneath the cerebral cortex, are primarily involved in motor control, and their lesions can present with psychiatric symptoms as well as differing degrees of cognitive dysfunction. Brainstem glioma is a very rare brain tumor that typically occurs in children and is infrequent in adults, representing only 1% to 2% of adult gliomas. The prognosis of brainstem gliomas is highly heterogeneous, with median survival times varying from 11 to 84 months (40), compared with the median survival time of 54 months in this study.

Glioma classification continues to evolve, and a new classification system based on key molecular parameters is of critical importance in providing diagnostic and prognostic information and optimizing tumor management. Generally, the higher the WHO grade of glioma is, the worse the prognosis. GBM is one of the most malignant central nervous system tumors and is classified as WHO grade 4. Common complications of GBM are depression and other mental diseases that may occur in conjunction with GBM; these are partly attributable to the negative prospects expected after a GBM diagnosis but may be due to the partially overlapping molecular mechanisms of the two diseases (10). Key molecular markers are indispensable and precise tools for improving diagnostic accuracy, determining prognosis and optimizing oncology risk management (41). However, some predictive models in our study did not include molecular indicators for the sake of model generalization. Enhanced detection of critical molecular parameters may help to construct models with satisfactory performance for supplying early and effective interventions and facilitating personalized treatment.

KPS is widely recognized as an effective and reliable tool for evaluating patients’ functional status (42) and has potential utility in predicting survival, assessing prognosis (43) and serving as an early predictor of glioma. Lower KPS is a risk factor for the incidence of PMHDs in glioma patients and negatively affects the survival time of patients (44). In this study, it was observed that the median survival time of the higher KPS group (KPS > 70) was longer than that of the lower KPS group (KPS ≤ 70), with significant statistical difference (22 vs. 66 months, *p* < 0.001). In addition, it should be recognized that there are varying degrees of correlation between variables. The presence of postoperative KPS may affect the significance of other variables in the prediction models, such as preoperative neurological deficits. Glioma patients with PMHDs often have longer hospitalization days, which may be related to their physical condition and severity of the disease. Furthermore, longer hospitalization days also mean increased costs and prolonged care support, resulting in greater financial and psychological burdens for patients and their families. Compelling evidence suggests that mental health disturbances carry substantial weight in the survival outcomes of glioma patients, as the log-rank test indicated that the survival curves are significantly different between the PMHD and control groups. Among all glioma patients, the median OS and 5-year survival rate were 19 months and 24.88% in the PMHD group, compared with 73 months and 53.51% in the control group, respectively. In GBM and DA patients with both WHO grade 4, the median OS was 13 and 14 months in the PMHD group and 23 and 46 months in the control group, respectively. In the PMHD group, the 5-year survival rate was 73.96% in LGG patients and 9.61% in GBM patients.

Nevertheless, several limitations of our study should be acknowledged. First, we mainly relied on simple indicators from the MDASI-BT to evaluate mental health disturbances in glioma patients without adopting international general scales, such as the Hospital Anxiety and Depression Scale (HADS), the Hamilton Anxiety Rating Scale (HAM-A) and the Positive and Negative Syndrome Scale (PANSS). In subsequent cohort studies, the application of generic scales and more extensive validation in the preoperative and postoperative follow-up were contemplated to evaluate patients’ mental health disturbances. Second, all patients were registered from only one hospital, and the establishment of a multicenter study with a larger sample size should be considered to minimize selection bias. Finally, this cohort study spanned over 8 years, and histological samples from patients with a relatively long history of glioma-related surgery could not be supplemented, making it impossible to retest some important molecular indicators. Moving forward, future studies should explore the comprehensive and detailed data of recent years based on the new WHO classification criteria to overcome this limitation.

In conclusion, potential predictors of PMHDs were identified, and the predictive models provide a new means of accurately assessing the underlying risks of PMHDs. These results may be used to assist clinicians with early intervention of possible mental health problems in glioma patients and to provide social and family support services targeted towards populations needing priority attention, thereby enhancing risk management, optimizing treatment strategies and improving prognosis. Elucidating the etiology and pathogenesis of PMHD, as well as avoiding exposure its risk factors, merits future endeavors.

## Data availability statement

The raw data supporting the conclusions of this article will be made available by the authors, without undue reservation.

## Ethics statement

The study protocol was approved by the ethics review committee of the Huashan Hospital Affiliated to Fudan University (receiving ethics committee number KY2015-256). Written informed consent was signed by all participants of the Central Nervous System Disease Tissue Bank at Huashan Hospital. The patients/participants provided their written informed consent to participate in this study.

## Author contributions

YW and JZ contributed equally to this work. Drafting of the manuscript: YW, JZ, and YY. Statistical analysis: YW and YY. Supervision: GQ and JW. Correspondence to: YY and JW. All authors contributed to the article and approved the submitted version.
