# Supplementary material for: Predictive models and survival analysis of postoperative mental health disturbances in adult glioma patients
Source: Front Oncol. 2023 Apr 21;13:1153455. doi: 10.3389/fonc.2023.1153455 (PMC10160603; doi:10.3389/fonc.2023.1153455)
Supplement: Supplementary file 1 [file Table_1.docx]

**Supplementary Online Content**

**‘Predictive Models and Survival Analysis of Postoperative Mental Health Disturbances in Adult Glioma Patients’**

**eTable 1.** Variance Inflation Factors for Different Logistic Regression Models Predicting Postoperative Mental Health Disturbances

**eTable 2.** The Simplified Model for Predicting Postoperative Mental Health Disorders in Glioma Patients

**eTable 1. Variance Inflation Factors for Different Logistic Regression Models Predicting Postoperative Mental Health Disturbances**

| **Preoperative Model of Glioma** | **VIF** | **Postoperative Model of Glioma** | **VIF** | **Postoperative Model of Glioblastoma, IDH-wildtype** | **VIF** | **Postoperative Model of Diffuse Astrocytoma, IDH-mutant** | **VIF** |
| --- | --- | --- | --- | --- | --- | --- | --- |
| Age (Years at Diagnosis) | 1.18 | Age (Years at Diagnosis) | 1.27 | Age (Years at Diagnosis) | 1.14 | Age (Years at Diagnosis) | 1.28 |
| Headache | 1.06 | Nausea | 1.02 | Headache | 1.32 | Illness Duration (Months) | 1.16 |
| Nausea | 1.03 | Intracranial Space-Occupying Lesion | 1.02 | Muscle Twitching | 1.41 | Headache | 1.35 |
| Intracranial Space-Occupying Lesion | 1.02 | Muscle Weakness | 1.09 | Muscle Weakness | 1.20 | Dizziness | 1.22 |
| Muscle Weakness | 1.09 | Limb Numbness | 1.05 | Limb Numbness | 1.14 | Epilepsy | 1.41 |
| Limb Numbness | 1.05 | Speech Disorder | 1.06 | Speech Disorder | 1.28 | Impaired Consciousness | 1.29 |
| Speech Disorder | 1.08 | Memory Deterioration | 1.06 | Memory Deterioration | 1.21 | Muscle Weakness | 1.48 |
| Memory Deterioration | 1.16 | Lethargy | 1.03 | Visual Impairment | 1.09 | Memory Deterioration | 1.22 |
| Slow Reaction | 1.17 | Corpus Callosum Glioma | 1.02 | Occipital Lobe Glioma | 1.22 | Visual Impairment | 1.20 |
| Lethargy | 1.02 | Basal Ganglia Glioma | 1.03 | Temporal Lobe Glioma | 1.20 | Parietal lobe Glioma | 1.22 |
| Temporal Lobe Glioma | 1.11 | Brainstem Glioma | 1.09 | Thalamic Glioma | 1.06 | Temporal Lobe Glioma | 1.29 |
| Corpus Callosum Glioma | 1.05 | WHO Grade | 1.72 | MGMT+ | 1.07 | Insular Glioma | 1.36 |
| Thalamic Glioma | 1.05 | Hospitalization Day | 1.06 | TERT+ | 1.06 | WHO Grade | 1.53 |
| Basal Ganglia Glioma | 1.05 | Chemotherapy/Radiotherapy | 1.12 | Hospitalization Day | 1.07 | Hospitalization Day | 1.21 |
| Brainstem Glioma | 1.02 | Karnofsky Score | 1.09 | Karnofsky Score | 1.25 | Karnofsky Score | 1.22 |

**eTable 2. The Simplified Model for Predicting Postoperative Mental Health Disorders in Glioma Patients**

| **Variables** | **Odds Ratio (95% CI)** | ***p* value** | **VIF** |
| --- | --- | --- | --- |
| **Preoperative Period** |  |  |  |
| **Age (Years at Diagnosis)** | 1.02 (1.01, 1.04) | 4.49×10^-4*^ | 1.22 |
| **Illness Duration (Months)** | 1.00 (0.99, 1.01) | 0.99 | 1.03 |
| **Epilepsy yes/no** | 0.76 (0.38, 1.53) | 0.44 | 1.06 |
| **Tumor Location (Involving Deep Structures) † yes/no** | 1.53 (1.08, 2.19) | 0.02^*^ | 1.05 |
| **Preoperative KPS** | 0.99 (0.97, 1.00) | 0.01^*^ | 1.02 |
| **Postoperative Period** |  |  |  |
| **WHO Grade** | —— | 0.07 | —— |
| **WHO Grade 2 (Ref)** | —— | —— | —— |
| **WHO Grade 1** | 1.24 (0.34, 4.50) | 0.74 | 1.12 |
| **WHO Grade 3** | 1.52 (0.88, 2.62) | 0.13 | 1.41 |
| **WHO Grade 4** | 1.74 (1.15, 2.61) | 0.01^*^ | 1.76 |
| **Hospitalization Day** | 1.02 (1.00, 1.04) | 0.02^*^ | 1.06 |
| **Chemotherapy/**  **Radiotherapy yes/no** | 0.71 (0.48, 1.06) | 0.09 | 1.13 |
| **Postoperative KPS** | 0.94 (0.93, 0.95) | 2.01×10^-40*^ | 1.08 |

†: Deep structures include deep brain locations such as the thalamus, corpus callosum, basal ganglia, brainstem, ventricles, and insula.

*: *p* < 0.05, **: *p* < 1.00×10^-4^.
